# Supplementary material for: Efficacy of Live Attenuated Vaccine and Commercially Available Lectin against Avian Pathogenic E. coli Infection in Broiler Chickens
Source: Vet Sci. 2020 May 13;7(2):65. doi: 10.3390/vetsci7020065 (PMC7355798; doi:10.3390/vetsci7020065)
Supplement: Supplementary file 1 [file vetsci-07-00065-s001.pdf]

Table S1. Weekly body weights, feed intake, body weight gain and feed conversion ratio of experimental groups.

Group [1]	Age [2]															
	Week 1				Week 2				Week 3				Week 4			
	BWT (g)	Feed intake (g)	BWT gain (g)	FCR	BWT (g)	Feed intake (g)	BWT gain (g)	FCR	BWT (g)	Feed intake (g)	BWT gain (g)	FCR	BWT (g)	Feed intake (g)	BWT gain (g)	FCR
**NC**	182 ± 12[a]	135	133	1.01	466 ± 30[a]	358	284	1.26	825 ± 66[a]	535	359	1.49	1326 ± 110[a]	751	501	1.50
**CBL-V**	183 ± 17[a]	138	134	1.03	475 ± 43[a]	365	292	1.25	841 ± 72[a]	534	366	1.46	1347 ± 113[a]	759	506	1.50
**CBL-V/O78-C**	183 ± 15[a]	136	133	1.02	479 ± 40[a]	373	296	1.26	810 ± 103[a]	490	331	1.48	1231 ± 94[ab]	716	421	1.70
**CBL-V/O125-C**	186 ± 13[a]	140	138	1.01	469 ± 38[a]	360	283	1.27	806 ± 88[a]	505	337	1.50	964 ± 149[abc]	395	158	2.50
**Lector+O78-C**	178 ± 14[a]	129	129	1.00	461 ± 34[a]	354	283	1.25	810 ± 79[a]	481	349	1.38	1227 ± 120[ab]	667	417	1.60
**Lector+O125-C**	179 ± 12[a]	131	131	1.00	463 ± 34[a]	359	284	1.26	857 ± 60[a]	539	394	1.37	1207 ± 138[ab]	543	350	1.55
**PC- O78**	184 ± 15[a]	137	137	1.00	465 ± 45[a]	353	281	1.25	789 ± 103[a]	495	324	1.53	870 ± 158[abcd]	324	81	4.00
**PC- O125**	177 ± 25[a]	130	130	1.00	466 ± 56[a]	364	289	1.26	851 ± 84[a]	578	385	1.50	940 ± 108[abc]	310	89	3.48

[1] Groups: NC; Negative control, CBL-V; vaccinated none challenged, CBL-V/O_{78}-C; Vaccinated challenged with *E. coli* O_{78}, CBL-V/O_{125}-C; Vaccinated challenged with *E. coli* O_{125}, Lector+O_{78}-C; Lector 3 days before challenge with *E. coli* O_{78}, Lector+O_{125}-C; Lector 3 days before challenge with *E. coli* O_{125}, PC- O_{78},; Positive control *E. coli* O_{78} challenge, PC- O_{125}; Positive control *E. coli* O_{125} challenge. [2] BWT; Bodyweight expressed as mean ± standard deviation, FCR; feed conversion ratio
